# Potential distribution dataset of honeybees in Indian Ocean Islands: Case study of Zanzibar Island

**DOI:** 10.1016/j.dib.2017.08.024

**Published:** 2017-08-30

**Authors:** Sizah Mwalusepo, Eliud Muli, Kiatoko Nkoba, Everlyn Nguku, Joseph Kilonzo, Elfatih M. Abdel-Rahman, Tobias Landmann, Asha Fakih, Suresh Raina

**Affiliations:** a icipe-African Insect Science for Food and Health, P.O. Box 30772, Nairobi, Kenya; b Department of General Studies, Dar es Salaam Institute of Technology, Dar es Salaam, Tanzania; c South Eastern Kenya University, P.O. Box 170-90200, Kitui, Kenya; d Department of Agronomy, Faculty of Agriculture, University of Khartoum, Khartoum North 13314, Sudan; e Agricultural Sector Development Programme, P.O.Box 159, Zanzibar, Tanzania

**Keywords:** Honeybees, Ecological modeling, Spatial distribution, Zanzibar Island

## Abstract

Honeybees (*Apis mellifera*) are principal insect pollinators, whose worldwide distribution and abundance is known to largely depend on climatic conditions. However, the presence records dataset on potential distribution of honeybees in Indian Ocean Islands remain less documented. Presence records in shape format and probability of occurrence of honeybees with different temperature change scenarios is provided in this article across Zanzibar Island. Maximum entropy (Maxent) package was used to analyse the potential distribution of honeybees. The dataset provides information on the current and future distribution of the honey bees in Zanzibar Island. The dataset is of great importance for improving stakeholders understanding of the role of temperature change on the spatial distribution of honeybees.

**Specifications Table**

**Table:** 

Subject area	Climate change and Biology
More specific subject area	Ecological modeling
Type of data	Maps and figures
How data was acquired	Field survey was carried out to collect presence data on honeybees in managed apiaries and wild nests across Zanzibar Island. Also, downscaled data of the Representative Concentration Pathways Scenarios, Fifth Assessment Report (RCPs-AR5) from AFRICLIM database was used to get temperature data in the sample locations.
Data format	Shape file (.shp) and raster
Experimental factors	We make use of AFRICLIM temperature dataset
Experimental features	The maximum entropy (Maxent) software version 3.3.3k and ArcMap version 10.1 which are geographic information system (GIS) and ecological niche modeling software, respectively were used for modelling and generating honeybees spatial distribution maps
Data source location	Unguja and Pemba Islands in Zanzibar
Data accessibility	Data are available in this article

**Value of the data**

• The data provide information on the potential spatial distribution of honeybees in both Unguja and Pemba Islands in Zanzibar and which is accessible for reuse.
• The data provide interesting and important information on future honeybee's distribution expansion in both Unguja and Pemba Islands in Zanzibar.
• The data can be used for modeling the effect of climate change and land use/ cover on honeybee's distribution in Indian Ocean Islands, in particular Zanzibar Island.
• The data can be useful for study genetic diversity of honeybees in Zanzibar Island.
• The data are important to different stakeholder include beekeeper communities, policy makers, international and local non-government organization engaged in apiculture intervention, researchers, scholars and academics.

## 1 Data

**Fig. 1:**
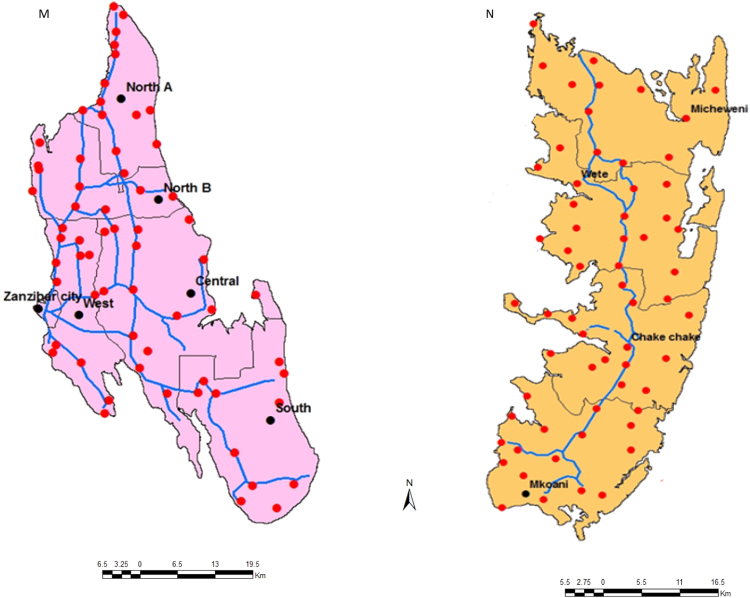
Study areas: (M) Unguja Island, (N) Pemba Island. The circular red dots indicate the location of honeybees occurrence records.

**Fig. 2:**
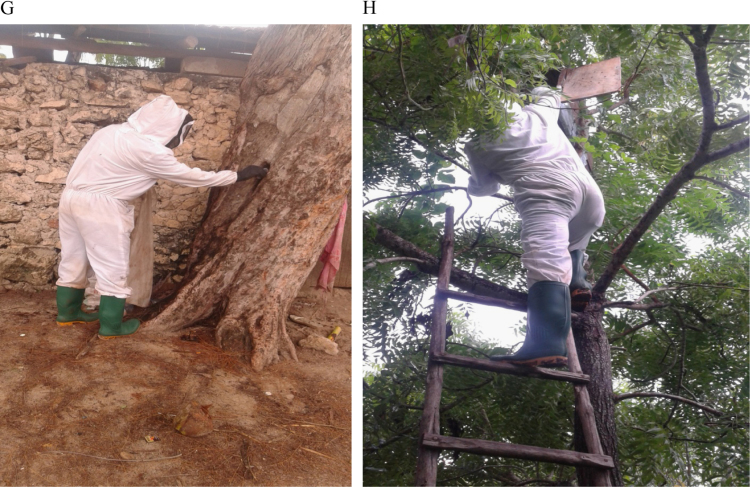
Honeybees data collection in a wild nest (G) and apiaries (H).

**Fig. 3:**
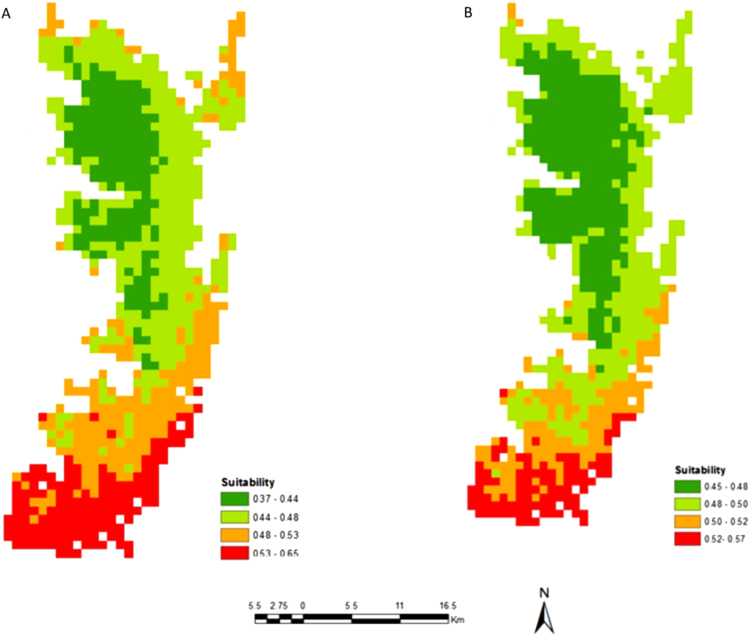
Probability of occurrence of honeybees in Pemba Island; under current (A) and future (B) temperature conditions.

**Fig. 4:**
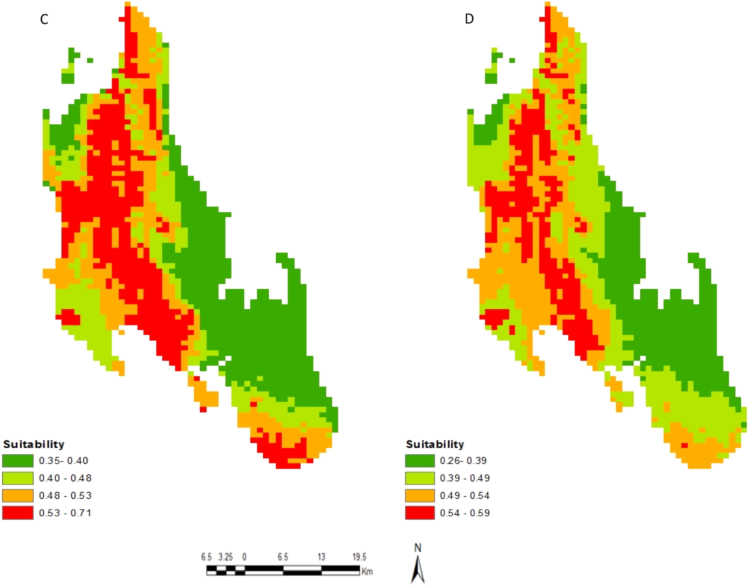
Probability of occurrence of honeybees in Unguja Island; under current (C) and future (D) temperature conditions.

Fig. 1 shows the study areas and locations of the occurrence records of honeybees in Unguja Island (M) and Pemba Island (N). Fig. 2 shows an example of honeybee's data collection in a wild nest (G) and apiaries (H). Probability of occurrence for honeybees according to temperature conditions in Pemba and Unguja Islands is shown in Fig. 3, Fig. 4, respectively. Under the current temperature conditions, the probability of occurrence shows that southern parts of Pemba have areas that are more suitable for honeybees than the northern parts in Pemba Island (Fig. 3A). In Unguja under current temperature conditions, the western parts and some parts of northern A and northern B of Unguja Island are more suitable compared to the southern and central parts (Fig. 4C). However, an area predicted as suitable does not mean that populations of the honeybee species will necessarily become successfully established there, but it is useful information for identifying areas of potential spread. The probability occurrence is predicted to decrease in both Islands in future by 2055 (Figs. 3B and 4D), probably due to temperature change that may cause further population decrease and extinctions of honeybees [1].

## 2 Experimental design, materials and methods

The dataset collected in this study is located in Zanzibar Island in Eastern and South Eastern coast of Africa [2]. The island is divided into two main islands namely; Unguja which is located between latitude 6°08´ Sand longitude 39°20´E, and Pemba Island which lies between latitude 5° 12′ S and longitude 39° 43′E. Field survey were carried out to collect honeybees occurrence data in managed apiaries and wild nests across Zanzibar in both Unguja and Pemba Islands (Fig. 1). A simple random sampling approach was used in the selection of apiaries and wild honeybees colonies for inspection. At each locality, the geographical coordinates (longitude and latitude) and altitude were recorded using a global positioning system (GPS). In total, 80 records per Island were collected. Data were checked for geographic accuracy using on screen visual inspection approach. Temperature data required to develop potential spatial distribution models of honeybees were obtained from AFRICLIM database (htt://www.york.ac.uk) of the Representative Concentration Pathways Scenarios, Fifth Assessment Report (RCPs-AR5) [3]. Downscaling of the data was obtained from regional climate models (RCMs), and clipped down in ArcMap (Version 10.1) software to Unguja and Pemba Islands. The maximum entropy (Maxent) software version 3.3.3k [4] was used for modelling honeybees’ spatial distribution. Models were calibrated using 75% of the records of honeybee's occurrence as training data, and the remaining 25% were used for validation.
